# *CHEK2* Germline Variants in Cancer Predisposition: Stalemate Rather than Checkmate

**DOI:** 10.3390/cells9122675

**Published:** 2020-12-12

**Authors:** Lenka Stolarova, Petra Kleiblova, Marketa Janatova, Jana Soukupova, Petra Zemankova, Libor Macurek, Zdenek Kleibl

**Affiliations:** 1 Institute of Biochemistry and Experimental Oncology, First Faculty of Medicine, Charles University, 12800 Prague, Czech Republic; lenka.stolarova@lf1.cuni.cz (L.S.); Marketa.Janatova@lf1.cuni.cz (M.J.); Jana.Soukupova@lf1.cuni.cz (J.S.); Petra.Zemankova@lf1.cuni.cz (P.Z.); 2 Laboratory of Cancer Cell Biology, Institute of Molecular Genetics of the Czech Academy of Sciences, 14220 Prague, Czech Republic; libor.macurek@img.cas.cz; 3 Institute of Biology and Medical Genetics, First Faculty of Medicine, Charles University and General University Hospital in Prague, 12800 Prague, Czech Republic; pekleje@lf1.cuni.cz

**Keywords:** checkpoint kinase 2, CHK2, CHEK2, KAP1, WIP1, germline mutation, hereditary cancer, breast cancer, prostate cancer, renal cancer, thyroid cancer, colorectal cancer

## Abstract

Germline alterations in many genes coding for proteins regulating DNA repair and DNA damage response (DDR) to DNA double-strand breaks (DDSB) have been recognized as pathogenic factors in hereditary cancer predisposition. The ATM-CHEK2-p53 axis has been documented as a backbone for DDR and hypothesized as a barrier against cancer initiation. However, although CHK2 kinase coded by the *CHEK2* gene expedites the DDR signal, its function in activation of p53-dependent cell cycle arrest is dispensable. *CHEK2* mutations rank among the most frequent germline alterations revealed by germline genetic testing for various hereditary cancer predispositions, but their interpretation is not trivial. From the perspective of interpretation of germline *CHEK2* variants, we review the current knowledge related to the structure of the *CHEK2* gene, the function of CHK2 kinase, and the clinical significance of *CHEK2* germline mutations in patients with hereditary breast, prostate, kidney, thyroid, and colon cancers.

## 1. Introduction

The accumulation of DNA mutations during the continually increasing human life span contributes to rising cancer prevalence worldwide [1]. Cancers are now the first or second leading cause(s) of premature death in individuals between 30 and 69 years in 91 countries of the world [2]. DNA alterations with increased cancer-promoting potentials affect tumor suppressor genes participating in DNA damage repair (DDR) and regulating cell cycle checkpoints [3,4]. Moreover, uncoupling these two processes may cause sustained proliferation of genetically unstable cells resulting in malignant transformation. 

Most cancers (over 90%) develop as sporadic tumors during life-long acquisition of DNA mutations. In contrast, less than 10% of cancers forms hereditary tumors that arise as a result of germline mutations in cancer predisposition genes [5]. Typical features—a high overall cancer risk, earlier age at disease onset, and 50% probability of transmitting the mutation to the offspring together with an accumulation of tumors in affected families—increase the medical importance of hereditary cancers and justify genetic counseling in affected families. Moreover, the share of hereditary tumors is higher in several frequent or highly malignant cancer types, including breast, pancreatic, or ovarian cancers. The identification of a causal mutation not only directs tumor-specific surveillance and preventive strategies but also impacts disease prognosis and targeted treatment [6]. In fact, proper identification and surveillance in mutation carriers has the potential to reduce the bulk of cancer-related mortality associated with several solid tumor types [7]. 

Fast progress in cancer genetics and the introduction of next-generation sequencing (NGS) have revolutionized the diagnostics of hereditary cancers in the last decade [8,9]. An analysis of individuals at risk using panels of cancer predisposition genes outperforms previous gene-by-gene analyses [10]. The availability of an easy and economically affordable panel NGS analysis together with the widening of testing criteria have brought high volumes of data. Germline variants found in cancer patients not only have confirmed the clinical utility of pathogenic mutations in high-penetrant “first wave” (including *BRCA1*, *BRCA2*, *MLH1*, and *MSH2*) and “second wave” (*PALB2*, *RAD51C*, and *RAD51D*) genes predisposing to common cancers but also have identified dozens of variants with unknown clinical significance (VUS) and variants of the “second wave” moderate penetrance genes (including *ATM* or *CHEK2*) [7,11]. While all cancer predisposition genes are now equal from the perspective of germline testing, the clinical utility of several genes (including *CHEK2*) varies in a broad interval delimited by their penetrance and population-specific prevalence [12]. The determination of penetrance requires careful assessment in the families of mutation carriers and in large populations of cancer patients and corresponding population-specific controls [13]. Moreover, the classification of germline variants and convincing identification of pathogenic mutations are demanding for most cancer predisposition genes.

In this review, we focus on the *CHEK2* gene coding checkpoint kinase 2 protein (CHK2), which was initially recognized as an effector kinase in the ATM-CHK2-p53 pathway in DDR, especially in response to DNA double-strand breaks (DDSB) [14,15,16]. The competence of the ATM-CHK2-p53 signaling cascade has been hypothesized as a barrier preventing early tumorigenesis [17], inducing cell cycle blockade, apoptosis, or senescence in transformed cells [18]. While initial studies associated *CHEK2* germline mutations with a moderate breast cancer risk, later ones identified a much wider portfolio of cancer types in *CHEK2* mutation carriers [19,20]. Routine genetic testing of *CHEK2* is now included in diagnostic NGS panels targeting various hereditary cancers, and *CHEK2* ranks among genes with the highest frequency of germline mutations. However, the presence of many variants of unknown significance (VUS) with a specific population prevalence prevents precise assessment of the risk associated with particular tumor types in *CHEK2* mutation carriers [21,22]. Thus, finding a *CHEK2* germline variant is sometimes perceived as a hindrance to a conclusive genetic interpretation rather than a gain for the clinical management of carriers. With this in mind, we have also reviewed the clinical importance of germline *CHEK2* mutations in patients with breast, prostate, kidney, papillary thyroid, and colorectal cancers.

## 2. Structure and Function of CHK2 Kinase

Human CHK2 kinase was identified in 1998 by Matsuoka et al. based on its homology to yeast checkpoint kinases Rad53 (in *Saccharomyces cerevisiae*) and Cds1 (in *Schizosaccharomyces pombe*). This pioneering work and subsequent papers from other laboratories were published with a short delay placed CHK2 downstream of ATM activation in DDR [23,24,25]. CHK2 kinase is widely expressed in proliferating, renewing cell populations but not in resting or terminally differentiated cells [26].

### 2.1. The CHEK2 Gene 

Tominaga and colleagues [27] localized the *CHEK2* gene to human chromosome 22 (22q12.1), where it spans 54 kb (chr22: 28,687,743–28,742,422; reverse strand; GRCh38). The most expressed transcription variant 1 (NM_007194/ENST00000404276.6) codes for an mRNA consisting of 15 exons with the translation start localized in exon 2. The relevance of alternative splicing variants remains unclear, but their proportion increases in tumor tissues [28]. Putative transcription factor binding sites (including SP1, CCAAT box, C/EBP, AP1, and E2F) were identified in the *CHEK2* promoter spanning the 268-bp region upstream of the transcription start site [29]. CpG islands identified in the 5′ region include a distal (and rarely methylated) CpG island (located −6000 to −8000 from ATG) and a proximal (heavily methylated) CpG island (located −300 to −600 from ATG) [30]. The 3′ portion of the *CHEK2* gene comprising exons 10–14 is duplicated with >90% homology in the human genome (on chromosomes 2, 7, 10, 13, 15, 16, 22, X, and Y) as non-expressed pseudogenes [31]. Munch and colleagues [32] performed a phylogenetic analysis of a *CHEK2* duplicon in anthropoids, indicating a burst of gene duplication in African great apes and humans.

### 2.2. Structure of CHK2 Kinase Protein

**Figure 1:**
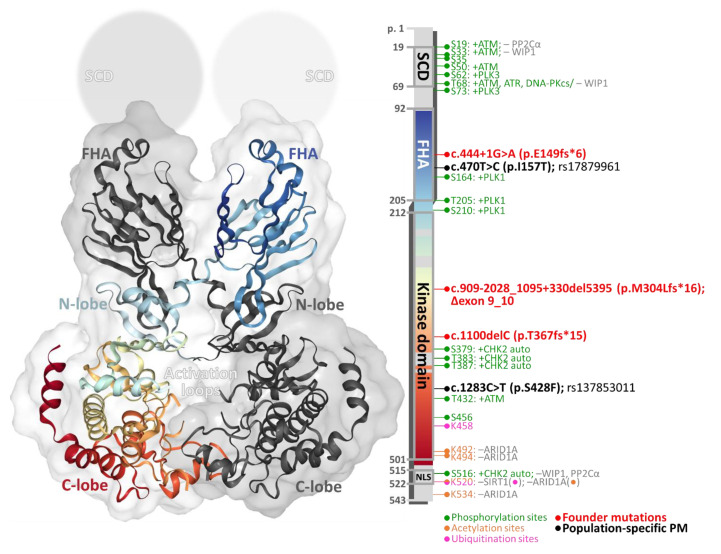
The structure of a CHK2 dimer (left; www.rscb.org/structure/3I6W) consists of intertwined monomers (one subunit is colored in gray, and the second subunit is colored in gradient from the N- to C-terminus; missing parts of 3D structures are colored in gray). The same color-coding of the bar (right) indicates the positions of conserved domains (boundaries reflect the crystallographic analysis by Cai et al. [35]). Lollipops depict known sites of covalent modifications, founder mutations, and variants.

The translation product of the dominant splicing variant consists of 543 amino acids forming the 65 kDa protein. CHK2 comprises three conserved functional domains including a SQ/TQ cluster domain (SCD) at the N-terminus, a forkhead-associated (FHA) domain, and a kinase domain (KD) at the C-terminus [33]. Crystallographic studies have unveiled a nearly complete CHK2 kinase structure in its monomeric and homodimeric forms with the exception of the SCD, extreme C-terminal parts, and several disordered regions, including activation loops (Figure 1) [34,35,36]. 

The SCD (residues 19–69) is characterized by seven pairs of serine–glutamine or threonine–glutamine (SQ/TQ) residues phosphorylated by ATM and other kinases [37,38]. It contains the T68 residue important for CHK2 activation; however, the entire SCD (51 amino acids) consists of 22 × S and 5 × T residues representing potential targets for other S/T protein kinases. The FHA domain (residues 92–205) is arranged in an 11-stranded β sandwich and mediates phosphorylation-dependent protein–protein interactions of CHK2 [34]. A seven-residue linker (residues 206–212) connects FHA and the kinase domain (KD). Almost half of the protein sequence comprises a serine–threonine KD (residues 212–501) consisting of two lobes forming an ATP-binding site at the cleft between them. The N-terminal lobe (residues 213–305) is formed mainly by β-sheet structures and contains a conserved E273 important for catalysis, while the larger C-terminal lobe (306–501) is mostly α-helical. The activation loop (residues 371–391) contains several activating phosphorylation sites (T383 and T387) that participate in substrate binding [35]. The nuclear localization signal (NLS) at the C-terminus is recognized by karyopherin-α2 (KPNA2) importing the CHK2 molecule into the nucleus [39]. 

### 2.3. Regulation of CHK2 Kinase Activity

Extensive covalent modifications of amino acid residues (phosphorylation, ubiquitination, and acetylation) and noncovalent interactions (homodimerization and phosphoprotein–protein interactions) influence the catalytic activity, substrate specificity, intracellular trafficking, and the half-life of CHK2 kinase.

#### 2.3.1. Phosphorylation

In the absence of DNA damage stimuli, CHK2 kinase resides in its monomeric inactive form. Upon DNA damage, ATM phosphorylates T68 [37] in the SCD of CHK2, promoting its transient homodimerization. ATM phosphorylation at the T68 priming site is important for full CHK2 activity in cells; however, CHK2 overexpression in bacteria or mammalian cells promotes dimerization and activation independently of ATM or ionizing radiation (IR) [40]. The exploration of CHK2 phosphorylation kinetics demonstrated that T68 phosphorylation occurs 3 min after neocarzinostatin treatment in HCT116 cells, followed by phosphorylation on S19 and S33/35 [38]. In addition, phosphorylation of other residues (S50 and T432) remains unclear [41,42]. Other candidate phosphorylation sites (S120, S260, T225, S379, and S435) were identified in recombinant CHK2 expressed in bacteria and insect cells expression systems using mass spectrophotometry, but their importance is largely unknown [43]. 

The process of CHK2 kinase activation includes the formation of a transient dimer through reciprocal FHA–KD and FHA–FHA interactions. The I157 residue resides in the center between these interfaces associating intramolecularly with the N-lobe in the KD [35]. The domain-exchanged, intertwined CHK2 homodimer promotes kinase activation by trans-autophosphorylation [35]. Wedged CHK2 molecules *trans-*phosphorylate T383 and T387 residues in activation loops exchanged between protomers (Figure 1), leading in turn to a disruption of homodimer conformation and release of the two catalytically active CHK2 monomers [34,35,36,44]. A description of another phosphoserine residue, S516, suggested that activating autophosphorylation of CHK2 can occur *in cis* (S516) and *in trans* (T383/387) depending on CHK2 dimerization [40,45]. The phosphorylation of CHK2 T68 has been frequently used as a marker of ATM activation [46], but T68 could be phosphorylated also by ATR in vitro [37] and by the DNA-dependent protein kinase catalytic subunit (PRKDC, alias DNA-PKcs) during mitosis [47]. Other CHK2 phosphorylation sites have been described as the targets of other kinases including polo-like kinase 3 (PLK3) [48,49] or PLK1 [50,51]. PLK3 phosphorylated S62 and S73 in vitro and was proposed to facilitate subsequent phosphorylation on T68 by ATM; however, this possibility has recently been challenged when no impact of PLK3 on checkpoint activation was found [48]. PLK1 in a complex with TP53-binding protein 1 (53BP1) phosphorylates CHK2 on S164, T205, and S210 to prevent its activation in mitosis, with S164 phosphorylation showing the greatest effect. Moreover, a co-localization of CHK2 with PLK1 has been observed during mitosis at centrosomes [52].

#### 2.3.2. Dephosphorylation

The activation of CHK2 in DDR is antagonized by its dephosphorylation by protein phosphatases, including WIP1 phosphatase (protein phosphatase Mg/Mn-dependent 1D; PPM1D) [53,54,55]. Human WIP1 belongs to a protein phosphatase type 2C family and is a homologue of Ptc2 and Ptc3, which regulate Rad53 in yeast [40,56]. WIP1 efficiently dephosphorylates residues at SQ/TQ sites in the CHK2 SCD, including T68 in a cell culture model; however, WIP1 is unable to dephosphorylate phosphorylated T387 in the activation loop [53]. It has been proposed that, under physiological conditions, this WIP1 activity participates in checkpoint recovery rather than in an inhibition of ATM/ATR-mediated response following DNA damage. It seems that fully active CHK2 kinase phosphorylated on residues in the activation loop is less sensitive to WIP1 dephosphorylation activity. Phosphorylated S516 is more accessible for dephosphorylation by other phosphatases, including PP2Cα [57]. Carlessi and colleagues [58] identified basal CHK2 phosphorylation (including T68) by tonic ATM signaling in undamaged cells and its counteraction by WIP1, PP2A, and PP1. The authors proposed that the activities of these phosphatases maintain the basal state of the ATM/CHK2 regulatory circuit. A recent study of clonal hematopoiesis in cancer patients treated by radiation, platinum, or topoisomerase II inhibitors found that preferentially selected somatic mutations affect all members of this circuit (*ATM*, *CHEK2*, *PPM1D*, and *TP53*) and increase the risk of therapy-related myeloid neoplasm development [59]. 

#### 2.3.3. Ubiquitination

CHK2 turnover is regulated by ubiquitin-mediated proteasomal degradation. Several E3 ubiquitin-protein ligases targeting CHK2 have been described. Ubiquitination catalyzed by the PIRH2 E3 ubiquitin-protein ligase (p53-induced protein with a RING-H2 domain) requires dephosphorylation of S456 in the CHK2 KD [60] and the presence of MDM2 (mouse double minute 2 homolog), an E3 ubiquitin-protein ligase, and P/CAF (p300/CBP-associated factor, known also as lysine acetyltransferase 2B; KAT2B), which was found to have an intrinsic E3 ligase activity [42,61]. Thus, phosphorylation at S456 increases CHK2 stability after DNA damage. In contrast, ubiquitination of CHK2 by seven in absentia homolog 2 (SIAH2) is independent of S456 phosphorylation and has been proposed as a mechanism regulating CHK2 basal turnover [62]. Ubiquitination of CHK2 catalyzed by the E3 ubiquitin-protein ligase complex containing Cullin 1 (CUL1) in response to DNA damage depends also on the autophosphorylation of S379 [63]. However, CUL1-mediated ubiquitination does not affect CHK2 stability. It has rather been proposed to contribute to CHK2-mediated apoptosis in U2OS cells in response to ionizing radiation. CHK2 has been identified in complexes targeted to DNA damage sites with a CHK2-interaction partner and adaptor protein MDC1 (mediator of DNA damage checkpoint protein 1) and with E3 ubiquitin-protein ligase RNF8; however, it has not been determined when CHK2 is ubiquitinated [63]. Recently, Wand and colleagues [64] described that ARID1A (AT-rich interactive domain-containing protein 1A), a component of SWI/SNF chromatin remodeling complexes, targets CHK2 for polyubiquitination at lysine residues K492, K494, K520, and K534. Thus, a loss of ARID1A by somatic mutations (ranking among the most frequent somatic alterations in various tumors) increases the CHK2 level. 

Ubiquitination is opposed by deubiquitinases. Among them, USP28 (ubiquitin-specific peptidase 28) and USP39 have been evidenced to deubiquitinate CHK2, with an apparent impact on CHK2 upregulation upon IR or cisplatin-induced DNA damage [65,66].

#### 2.3.4. Acetylation

Although acetylases modifying CHK2 by acetylation are largely unknown, several reports have described not only CHK2 deacetylation catalyzed by NAD+-dependent histone deacetylase SIRT1 targeting histones but also non-histone proteins implicated in the regulation of many physiological and pathological processes, including DDR and tumorigenesis [67]. A recent study showed that SIRT1 directly deacetylates K520 in CHK2, suppressing its phosphorylation, dimerization, and thus activation. Moreover, this study provides evidence that Chk2 hyperactivity in Sirt1−/− mice is responsible for embryonic lethality that could be rescued by Chek2 co-deletion. 

In conclusion, CHK2 covalent modifications affect its catalytic activity, turnover, and targeting. However, it is still not certain how covalent modifications influence CHK2 substrate specificity and direct CHK2 functions depending on the cell cycle phase.

### 2.4. CHK2 Substrates and Its Effector Pathways

Once activated, CHK2 phosphorylates many intracellular targets carrying a consensus motif containing a hydrophobic amino acid (B^α^) at position −5 and arginine (R) residue at position −3: B^α^-X-R-X-X-S/T [68]. Activated CHK2 kinase then participates in the regulation of many intracellular pathways that were comprehensively reviewed by Zannini and colleagues [69]. Our review summarizes CHK2 activities related to the process of tumorigenesis.

#### 2.4.1. CHK2 in the Regulation of the Cell Cycle, Apoptosis, and Senescence

**Figure 2:**
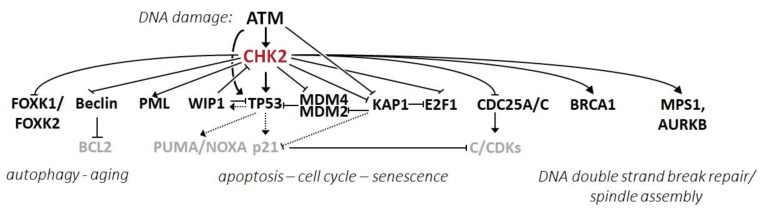
In the presence of DNA damage, especially in the presence of DNA double-strand breaks, sensor protein complexes (not shown) activate the apical kinase ATM phosphorylating CHK2. As an effector kinase, CHK2 phosphorylates numerous substrates (described in the text) participating in critical pathways deregulated in the process of tumorigenesis.

CHK2 is traditionally portrayed as an effector kinase in the ATM-CHK2-p53 pathway mediating response to DDSB (Figure 2) [14,15,16]. The double-strand DNA breaks, representing highly toxic events in proliferated cells, are recognized by the MRE11-RAD50-NBN1 (MRN) complex recruiting ataxia telangiectasia-mutated (ATM) kinase into the site of DNA damage [70]. This, in turn, activates ATM, a master regulator of the DDR [71]. ATM phosphorylates CHK2 and other proteins orchestrating DDSB repair and DDR. CHK2 overlaps some ATM targets, amplifies an ATM-triggered signal, and increases the DDR regulation fidelity. A parallel DDR pathway, ATR-CHK1-p53 exploiting CHK1 kinase, is activated mainly by the presence of long stretches of single-stranded (ss)DNA or DNA crosslinks, and it targets several overlapping substrates [72,73].

One of the first reported CHK2 targets is the tumor suppressor protein p53. Activated ATM phosphorylates p53 at S15, increasing its stability and activation [74]. Simultaneous phosphorylation of MDM2 disrupts MDM2–p53 interaction and allows p53 stabilization. CHK2 acts on p53 in a similar fashion and phosphorylates p53 at S20, contributing to p53-dependent cell cycle arrest in the G1 phase [75]. Besides, CHK2 phosphorylates MDM4 on S342 and S367 in vivo [76], possibly impacting the transcriptional activity of p53. p53 transactivation targets include the *CDKN1A* gene, coding the inhibitor of cyclin/CDK complexes p21^CIP1/WAF1^ and the *GADD45A* (growth arrest and DNA damage inducible α) gene coding a versatile stress sensor [77,78,79]. Although CHK2 was initially implicated in the induction of a p53-dependent checkpoint, more recent studies have suggested that the endogenous level of CHK2 does not cause G1 arrest and that the observed checkpoint activation may be an artefact attributable to CHK2 protein overexpression [80]. In agreement with this, no checkpoint defect was observed in HCT116 cells lacking CHK2 and thus the impact of CHK2 on the p53 pathway remains unclear [33,81].

The expressions of p21 and GADD45a are also inhibited by the KRAB (Kruppel-Associated Box Domain)-Associated Protein 1 (KAP1) transcription co-repressor targeting the KRAB-zinc finger protein superfamily of transcription factors [82]. Upon DNA damage, KAP1 is phosphorylated by ATM and CHK2 (or CHK1) at S824 and S473, respectively [83]. While CHK2 phosphorylates KAP1 dominantly in an etoposide- or ionizing radiation-induced stress response, CHK1 targets the same S473 residue in response to UV radiation. KAP1 S473 phosphorylation relieves its transcriptional repression, which results in increased p21 and GADD45 expressions at the G2/M checkpoint [84]. However, KAP1 S473 also provides a binding site for the E2F1 transcription factor involved in the cell cycle and apoptosis. Increased interaction between KAP1 phosphorylated at S473 and E2F1 decreases the expression of a subset of proapoptotic genes and apoptosis [83]. Besides, CHK2 directly phosphorylates E2F1 at S364, which results in increased E2F1 protein stability and transcriptional activity towards p53-independent apoptosis [85]. Thus, CHK2-activated KAP1 phosphorylation may counteract CHK2-induced E2F1 activity in DDR as a negative regulatory feedback mechanism. Targeting this regulatory network by combination chemotherapy using etoposide and inhibitors of KAP1-S473 phosphorylation may potentiate the cytotoxic effect of chemotherapy [83].

CHK2 has been shown to phosphorylate CDC25 phosphatases, a family of homologous dual-specific enzymes dephosphorylating inhibitory phosphothreonine or phosphotyrosine residues on cyclin-dependent kinases (CDKs) stimulating transition through the cell cycle [86]. The inhibition of CDC25 phosphatases by phosphorylation ensures rapid but transient checkpoint activation, while the activation of p53 is required for longer cell cycle arrest, the induction of senescence, or apoptosis [87]. A CHK2-mediated phosphorylation of CDC25A phosphatase at S123 inhibits dephosphorylation of the cyclin-dependent kinase 2 (CDK2)-cyclin E complex, halting the cell cycle before entry into the S phase [14]. Moreover, CHK2 also phosphorylates CDC25C phosphatase at S216, stimulating an interaction of CDC25C with 14-3-3 proteins. The interaction with 14-3-3 proteins displaces CDC25C from binding and the dephosphorylation of a CDK1-cyclin B complex, required for its activation before mitotic entry [16,88]. 

CHK2 phosphorylates S117 of the promyelocytic leukemia protein (PML, a tumor suppressor involved in multiple apoptotic pathways) and increases its activity in the induction of γ-radiation-induced apoptosis [89]. In contrast, a fusion protein of PML with retinoic acid receptor α (PML-RARα), resulting from frequent translocation in acute promyelocytic leukemia (t15;17), suppresses CHK2 and inhibits its autophosphorylation [90,91].

#### 2.4.2. CHK2 in the Regulation of DNA Repair and Mitotic Spindle 

In response to ionizing radiation, CHK2 phosphorylates breast cancer susceptibility protein 1 (**BRCA1**) at S988, which is believed to modulate the BRCA1 function in DNA repairs towards a homologous recombination (HR) repair instead of non-homologous end joining (NHEJ) [92,93]. Alongside its function in HR, BRCA1 (and other proteins identified as regulators or executors in DDR) has been implicated in mitotic spindle assembly [94]. In case of spindle damage, BRCA1 gets phosphorylated at S988 by CHK2, which leads to protein accumulation and to inhibition of the microtubule-nucleating activity of the centrosome [95,96,97]. The activity of CHK2 in centrosome regulation includes also the phosphorylation of S/T residues in other regulators, including T288 in the dual specificity protein kinase TTK (alias MPS1), S331 in aurora kinase B (AURKB), or S507 in myosin phosphatase targeting subunit 1 (MYPT1) [94,98]. Cells and organisms lacking CHK2 are viable and fertile, suggesting that its function in mitosis is not essential, and thus, the precise impact of CHK2 on cell division remains to be elucidated.

#### 2.4.3. CHK2 in the Regulation of Autophagy and Aging 

CHK2 kinase was also reported to be involved in other processes apart from DDR or cell cycle regulation. In response to oxidative stress, CHK2 has been linked to cell protection via autophagy. High levels of reactive oxygen species (ROS) and hypoxia were reported to trigger the ATM-CHK2 axis and the phosphorylation of Beclin 1 [99]. Beclin 1, coded by the tumor suppressor gene *BECN1*, is an essential regulator of autophagy, and its phosphorylation at S90/S93 by CHK2 has been shown to disrupt the formation of Beclin 1 (BCL2 autophagy-regulatory complex), reducing ROS production by the autophagy of damaged mitochondria. Thus, the ATM-CHK2-BECN1 autophagy axis may serve as a physiological pathway preventing tissue damage following ischemia [99]. In addition, CHK2 phosphorylates Forkhead transcription factors FOXK1 and FOXK2, which act as transcriptional repressors of autophagy-related genes [100]. CHK2-mediated FOXK phosphorylation induces their binding to 14-3-3 proteins, which, in turn, traps FOXK in the cytoplasm and induces autophagy following DNA damage. 

#### 2.4.4. CHK2 in the Regulation of Other Intracellular Pathways 

While CHK2 kinase was characterized as a downstream kinase transmitting DDR signal onto effectors over 20 years ago, new functions of CHK2 and the ATM-CHK2 axis have been identified and reviewed by Zannini and colleagues [69]. These “non-canonical” CHK2 activities include stem cell maintenance, regulation of the intracellular response to a viral infection, or the participation of circadian clock regulation. 

Despite substantial progress, it should be noted that many canonical as well as novel CHK2 functions have been studied dominantly in model systems involving tumor cell lines. However, little is still known about the real demand for CHK2 functions in particular tissues under physiological and pathological conditions. Animal experiments with *Chek2* knockout (*Chk2*^-/-^) mice demonstrated that *Chk2*^-/-^ mice are viable and fertile, developing a slightly increased tumor incidence with a long latency, and that they are more radioresistant compared with wild type Chk2 mice [101]. This indicates that Chk2 activity is redundant and may be compensated for example by Chk1 kinase sharing overlapping substrates. This hypothesis has supported subsequent experiments demonstrating that double mutant *Chk1*^+/-^/*Chk2*^-/-^ and *Chk1*^+/-^/*Chk2*^+/-^ mice have a progressive cancer-prone phenotype [102]. 

Interestingly, the prevalence of germline *CHEK2* mutations in cancer patients outnumbers that in *CHEK1* by the order of magnitude. The same is true also for somatic mutations in these two kinases. Individuals carrying bi-allelic *CHEK2* mutations have a normal phenotype; however, they carry an increased cancer risk in comparison with heterozygotes and noncarriers [103,104]. However, the cell-type specific demand for CHK2 activation in human tissues is largely unknown. Recently, van Jaarsveld and colleagues [105] compared CHK2 activation in primary breast and lung cells, describing a significantly higher CHK2 activity in breast than in lung primary cells. These observations can further stimulate investigations revealing tissue-specific cancer development in *CHEK2* mutation carriers.

## 3. Germline *CHEK2* Variants 

Germline mutations in the *CHEK2* gene and their association with cancer development were originally described in 1999 (a year after its discovery) by Bell and colleagues [19], who identified the most studied population-specific *CHEK2* variants c.1100delC and p.I157T in predominantly breast cancer patients from p53-wild type Li-Fraumeni syndrome (LFS) and LFS-like (LFL) families. The observation that *CHEK2* mutations associate with these clinically severe syndromes alongside the functional activity of CHK2 kinase in DDR attracted huge interest, and for a while, *CHEK2* was a candidate for the putative “*BRCA3*” gene. The first functional analysis revealed that c.1100delC completely abrogates CHK2 kinase activity [106]. However, the association between germline *CHEK2* mutations and LFS/LFL was disputed soon afterwards [31,107,108,109,110,111]. Moreover, the CHEK2 consortium (comprising laboratories from the UK, the Netherlands, the USA, and Germany) identified only an incomplete segregation of c.1100delC with cancer phenotypes in breast cancer families [112] and found a high prevalence of heterozygous c.1100delC carriers, exceeding 1% in Netherlands and UK controls. The high prevalence of c.1100delC in northern Europe was also confirmed by a study from Finland [110]. Although these early studies found that the frequency of c.1100delC mutations was enriched among breast cancer patients with early and/or double primary tumors and in multiple cancer families, an incomplete penetrance of c.1100delC in cancer families and a high prevalence of the variant in controls substantially distorted *CHEK2′*s credit as a clinically considerable predisposing gene. In contrast, Cybulski and colleagues [20] analyzed two founder truncations, c.1100delC and c.444+1G>A, and the p.I157T missense variant in a large group of Polish patients and characterized *CHEK2* as a multi-organ cancer susceptibility gene. Their analysis of 4008 cases with 13 tumor types and 4000 controls found a moderately increased risk of breast, prostate, and thyroid cancer in carriers of truncating *CHEK2* mutations and an increased risk of breast, colon, kidney, prostate, and thyroid cancer for the carriers of p.I157T. Since then, a growing body of evidence has suggested that germline *CHEK2* variants deserve interest from the perspective of clinical oncology as their carriers face an increased risk of various cancer types that display some specific clinicopathological characteristics.

Unfortunately, the identification of c.1100delC (and a few other variants) as a *CHEK2* founder mutation (Figure 1) limited *CHEK2* analyses dominantly to these variants in most pre-NGS studies. Aloraifi and colleagues performed a meta-analysis of protein-truncating variants in moderate-risk breast cancer genes in 2015 and cited only 12 out of 54 published *CHEK2* analyses (22%) that had performed full gene scanning [113]. Recently, a spectrum of *CHEK2* pathogenic/likely pathogenic variants identified in 2508 carriers analyzed by GeneDx in the USA was published by Sutcliffe and colleagues [114]. They showed that nearly 95% of all carriers have some of the 18 variants detected more than 10 times, while the remaining approximately 5% of individuals carried one of the 101 rare germline variants including 17 large intragenic rearrangements. About 73% of individuals carried some of the five most frequent founder variants (including p.I157T and p.S428F). A full gene analysis was largely introduced with NGS panels. However, the identification of copy number variations (CNV), which represent a substantial fraction of *CHEK2* germline mutations (exon 9–10 deletions (denoted also 5395del) in Slavic populations [115] and US patients [114] and exons 2–3 and 6 in Greece [116]), is still not a golden standard. Besides, pseudogene sequences homologous to exons 10–14 limited analyses in early NGS studies [117,118]. Thus, our understanding of *CHEK2′*s contribution to cancer predisposition is incomplete as founder mutations vary among different ethnics and non-founder alterations account for over 25% of *CHEK2* pathogenic variants.

The bottleneck limiting the clinical outcomes of NGS analyses is rare VUS [119]. They currently account for 1228 out of 2195 (55.9%) germline *CHEK2* variants reported in ClinVar (https://www.ncbi.nlm.nih.gov/clinvar/?term=chek2; accessed 07-11-2020). As the majority of the *CHEK2* coding sequence comprises established domains, the prioritization of *CHEK2* VUS based on their presence in conserved regions is useless. Some studies have aimed to perform functional analyses challenging the catalytic activity; the activities of putative substrates; protein stability; and dimerization or localization of investigated CHK2 isoforms using in vitro [120,121], bacterial [122], yeast [21,123,124,125], or human cell models [115]. Besides a handful of exceptions [21,115,123], however, the published studies have only analyzed a single or a few variants, and their results were mutually concordant only in part. Therefore, a systematic analysis of *CHEK2* VUS is highly desirable as rare missense variants or small in-frame deletions are frequent and they may represent 25–50% of all germline *CHEK2* alterations [114,115,122,126,127].

### 3.1. Ethnic and Geographical Differences in CHEK2 Mutation Frequency

The prevalence of germline *CHEK2* variants substantially varies among different populations and ethnics. These differences can be demonstrated on multiethnic studies utilizing an identical approach. Kurian and colleagues collected data from germline testing in 5900 breast and 937 ovarian cancer patients from California and Georgia [128]. They found that pathogenic *CHEK2* variants were the third most frequent germline alterations in both cancers (following *BRCA1/BRCA2* variants); however, *CHEK2* significantly prevailed in whites over blacks in both breast cancer (2.3% vs. 0.15%) and ovarian cancer (1.3% vs. 0%). An analysis by Caswell-Jin et al. also identified significant differences in the frequency of pathogenic *CHEK2* mutations between whites and non-whites (3.8% vs. 1.0%; *p* = 0.002) tested for hereditary cancer risk [22]. 

Although *CHEK2* has the highest mutation prevalence among Caucasian individuals of European descent, the spectrum and frequency of founder as well as non-founder mutations vary among particular European populations. The frequency of the European founder mutation c.1100delC declines from the north to the south [129], with carrier frequency in the general population close to 1% in the UK and the Netherlands but very rare in the Mediterranean region [130,131,132]. The most frequent European *CHEK2* variant, p.I157T, has a population frequency of heterozygous carriers of around 5% in Poles [20], Latvians [133], Hungarians [134], and Russians [135] and around 2–3% in Czechs [136], Slovaks [134], and Germans [126]. Interestingly, the p.I157T allele has developed in some populations independently [137]. This high population frequency rules out the possibility that the p.I157T variant could have a higher than low impact on cancer susceptibility; however, an increased risk with odds ratio (OR) approximately 1.5 in p.I157T carriers has been described systematically in case control studies and meta-analyses for breast cancer (Table 1) and other cancer types. Another central European founder mutation, a deletion of exons 9–10, was described by Walsh et al. [138] in patients of Czech and Slovak origins (Figure 1). A high background frequency of this variant in controls was also found in Poland (0.4%) [139] and Latvia (0.7%) [140]. 

The lowest frequency of *CHEK2* germline mutations is reported in patients of Asian origin. A panel NGS analysis involving 8085 Chinese breast cancer patients revealed only 18 (0.3%) carriers of pathogenic *CHEK2* mutations [141]; eight of them carried the novel founder nonsense mutation c.C417A (p.Y139*) [142]. Only two carriers (0.24%) of *CHEK2* mutations were identified in a recent analysis of 831 breast cancer patients from Shanghai [143]. Studies of breast and prostate cancer patients from Japan included analyses in control populations that revealed the presence of pathogenic *CHEK2* germline mutations in 0.1% of both female and male noncancer controls [144,145].

### 3.2. Breast Cancer

Most studies of *CHEK2* germline mutations have dealt with breast cancer patients. The estimated OR for carriers of *CHEK2* mutations varies among the studies considerably depending on analyzed populations, CHEK2 variants, and used controls (Table 1).

The variability of risk estimates is influenced by several important parameters, including the number and (pre)selection criteria of eligible patients, *CHEK2* variants analyzed and considered pathogenic, and control group selection. The estimated lifetime risk of breast cancer for *CHEK2* mutation carriers (mostly c.1100delC) differs according to family cancer history and ranges from 20 to 40% in women without and with a positive family breast cancer history, respectively [150]. More specifically, Cybulski and colleagues [159] estimated the lifetime BC risk for truncating *CHEK2* mutations in Polish patients to be 20% in women without a family cancer history and 28% and 34% in women with a second- and first-degree relatives with BC, respectively. A Danish case-control study determined an absolute 10-year BC risk as 24% in women carrying c.1100delC and older than 60 years undergoing hormone replacement therapy (HRT) with BMI > 25 [162]. Johnson et al. [177] estimated a cumulative risk of 58.8% (95% CI 33.8–85.3) for breast cancer by the age of 80 for first-degree relatives of c.1100delC carriers with bilateral breast cancer from the UK. An international European study predicted the lifetime risk for BC in daughters of c.1100delC carriers and noncarriers with bilateral breast cancer as 37% and 18%, respectively [161]. It can be assumed that breast cancer risk associated with pathogenic *CHEK2* variants in the general population would be at the lower moderate penetrance gene border (OR > 2) but considerably higher (though still in a moderate penetrance range; with OR < 4) for high-risk carriers from families with a positive cancer history. A precise evaluation of the associated risk will require large studies of unselected cancer patients with an appropriately selected population of geographically matched controls. A more precise estimate of individual breast cancer risks associated with germline *CHEK2* mutations could be reached by considering the polygenic risk score (PRS) [178,179,180].

Breast cancer in the carriers of pathogenic germline *CHEK2* mutations has several recurrently reported clinicopathological characteristics. The most striking is the development of bilateral breast cancer, as shown in some studies (Table 1). A recent meta-analysis by Akdeniz and colleagues [181] computed the relative risk of contralateral breast cancer development as 2.68 (95% CI 1.69–3.65) for c.1100delC mutation carriers versus noncarriers (which was fully comparable with that in *BRCA2* mutation carriers: RR = 2.75; 95% CI 1.77–4.29). A significantly younger cancer onset in *CHEK2* mutation carriers has been reported less consistently [152,154,182]. Published studies have also pointed out a worse breast cancer prognosis for c.1100delC mutation carriers [183,184,185,186] but not for p.I157T carriers [187]. Since the first studies, *CHEK2* germline mutations have frequently been associated (in 85–90% of cases) with estrogen receptor positive (ER+) breast cancer subtypes [115,126,139,183,188,189]. Consistent with that, no *CHEK2* mutation carriers were observed in an analysis of 1824 triple negative breast cancer patients [190]. A large analysis conducted by the BCAC consortium estimated the cumulative risk of developing ER+ and ER− breast cancer by the age of 80 for c.1100delC mutation carriers at 20% and 3%, respectively, compared with 9% and 2%, respectively, in the general British population [155].

Although ER+ tumors tend to have a better prognosis in unselected breast cancer patients, ER+ tumors in *CHEK2* mutation carriers were associated with worse breast cancer-specific survival [155,184]. A low or significantly reduced CHK2 expression was found in most breast tumors from mutation carriers [191]. Interestingly, both tumors with low CHK2 expression and tumors from CHEK2 mutation carriers were associated with increased grade, especially with a lower proportion of grade one tumors [115,192]. Bahassi and colleagues [193] offered an interesting hypothesis describing a link between ER positivity and reduced CHK2 expression based on the observation of mouse models. They noticed that the ER stimulated c-MYC transcriptional activity, increasing CDC25A expression that in turn resulted in the S-phase entry and genomic instability in mice homozygous or heterozygous for Chk2 c.1100delC. An association with lobular breast cancer was reported for p.I157T in patients from Poland [194] and the Czech Republic [115] and from a meta-analysis by Liu and colleagues [173] for pathogenic *CHEK2* mutations in patients from Slovenia [182] and for other germline variants in patients from Bulgaria [195].

All in all, germline *CHEK2* mutations confer increased risk of the development of ER-positive breast cancer with an unfavorable prognosis and an increased risk of bilateral breast cancer. The current NCCN guidelines (National Comprehensive Cancer Network guidelines version 1.2021 for Genetic/Familial High-Risk Assessment: Breast, Ovarian, and Pancreatic) recommend annual mammogram screenings for women carrying a pathogenic mutation since the age of 40 and recommend an annual MRI check. The risk-reducing mastectomy (RRM) is not generally recommended because of the lack of data confirming its benefits; however, RRM can be considered, especially based on family cancer history [196]. Prophylactic contralateral mastectomy can also be recommended for breast cancer patients with pathogenic *CHEK2* germline mutations. Chemoprevention for unaffected women with pathogenic mutations could be considered as an option [197]. 

An increased risk of male breast cancer has been documented in few smaller studies (Table 1); however, due to the low overall male breast cancer risk, its increase would not substantiate a specific follow-up, although the association should be considered in case of breast pathology developing in male mutation carriers. The RRM is not recommended in male *CHEK2* mutation carriers [196]. 

The relatively high frequency of germline *CHEK2* mutations in some populations and the dispensability of CHK2 for normal development results in identification of recessive homozygotes or compound heterozygotes carrying *CHEK2* mutations at both alleles. Sutcliffe and colleagues reported 32 (1.3%) homozygotes among 2508 identified *CHEK2* mutation carriers [114]. The most frequent ones were c.1100delC and p.I157T homozygotes, of whom 66% and 60% were diagnosed with BC, respectively. Rainville summarized data from 31 biallelic mutation carriers identified among 6473 monoallelic *CHEK2* mutation carriers tested by Myriad Genetics, of whom 16/31 were c.1100delC mutation carriers [104]. Compared with monoallelic carriers, biallelic carriers developed breast cancer more frequently (81% vs. 41%; *p* < 0.0001) and more likely before the age of 50 (61% vs. 24%; *p* < 0.0001), they developed secondary breast cancer with a higher frequency (23% vs. 8%; *p* = 0.01), and finally they had a higher risk of developing any primary cancer and multiple primary cancer. The ORs for the development of ductal carcinoma *in situ* (DCIS) and ductal carcinoma were high (OR 8.7, 95% CI 3.7–20.5 and OR 5.0, 95% CI 2.0–12.4, respectively) [104]. Case reports of homozygous carriers, which included other *CHEK2* mutations, have been published episodically [103,198,199], and they indicate an increased risk of the development of variable primary cancers with an early age at onset. These data support an intensified management of homozygous carriers of *CHEK2* pathogenic mutations. In the case of breast cancer, the surveillance follow-up should copy that in *BRCA1/BRCA2* mutation carriers. The prevention of other tumors associated with germline *CHEK2* mutations should be considered. While p.I157T is considered a low penetrance variant, we assume that, based on functional data, homozygotes should be managed in a way similar to c.1100delC heterozygotes [115].

### 3.3. Prostate Cancer

The association of *CHEK2* with prostate cancer was already proposed in 2003, when Dong and colleagues identified 18 unique *CHEK2* mutations in 15/400 (3.75%) patients with sporadic prostate cancer and in 11/298 (3.69%) patients with familial prostate cancer [200]. This association has been confirmed by subsequent studies (Table 2), and a *CHEK2* gene analysis is currently routinely performed as part of prostate-specific gene panels [201].

A study by Pritchard et al. demonstrated that 82/692 (11.8%) men with metastatic prostate cancer carry a mutation in some of the 16 analyzed DNA repair genes. The carriers of *BRCA2* (N = 37; 44%), *ATM* (N = 11; 13%), *CHEK2* (N = 10; 12%), and *BRCA1* (N = 6; 7%) represented over 75% of all mutation carriers. These data confirmed a report by Isaacsson Velho and colleagues [209], who found a similar proportion of mutation carriers (21/150; 14%) among unselected prostate cancer patients with an identical proportion of affected genes: *BRCA2* (N = 9; 43%), *ATM* (N = 3; 14%), *CHEK2* (N = 3; 14%), and *BRCA1* (N = 2; 9%). Moreover, patients with germline mutations had significantly more frequent intraductal histology (47.6% vs. 11.6% in noncarriers; *p* = 0.003) and presence of lymphovascular invasion (52.3% vs. 13.9%; *p* < 0.001). Thus, the authors concluded that genetic testing should be offered to patients with these clinicopathological characteristics. A larger cross-sectional analysis of 1328 men with prostate cancer by Giri and colleagues [210] found 15.6% carriers of germline mutations; 10.9% patients carried a mutation in DNA repair genes (*BRCA2* > *CHEK2* > *ATM* > *BRCA1*), increasing the risk of more advanced tumors (Gleason score ≥ 8). *CHEK2* mutations were less frequent in prostate cancer patients from Japan [144].

Wu and colleagues analyzed survival characteristics in prostate cancer patients carrying germline *CHEK2* mutations [211]. Although they found no association between the *CHEK2* mutation status and early diagnosis or PrC, they noted that c.1100delC mutation carriers are more prevalent among patients with a lethal disease than in patients with low-risk prostate cancer (1.28% vs. 0.16%; *p* = 0.004). Yadav et al. [212] found no significant association between the presence of germline mutations and survival characteristics, but they found that mutations in *ATM*, *BRCA2*, *CHEK2*, *FANCM*, and *TP53* were significantly more frequent in patients with a metastatic disease.

The performed studies present rather compelling evidence that *CHEK2* is a low-to-moderate prostate cancer predisposition gene. Therefore, male carriers of pathogenic *CHEK2* mutations, especially from families with multiple prostate cancers, deserve intensified prostate cancer screening which should include an annual PSA test from the age of 40. A report by Cybulski et al. [213] identified an increased proportion of p.I157T carriers among individuals with elevated PSA or an abnormal digital rectal examination versus individuals with normal assessments (10.2% vs. 4.3%; OR = 2.5; *p* = 0.0008); however, a prostate-specific follow-up needs to be justified in larger studies. 

### 3.4. Kidney Cancer

The p.I157T variant (but not the truncating founder mutations) was recognized to increase kidney cancer risk in a pioneering clinical study by Cybulski and colleagues [20]. Other studies (Table 3) confirmed an association between *CHEK2* germline mutations and renal cell carcinoma later on, with the exception of an analysis by Ge et al. exploiting GWAS datasets.

NGS panel sequencing by Carlo and colleagues in 254 patients with advanced RCC identified 41 carriers of pathogenic germline mutations in renal cancer- or other cancer-associated genes [215]. Among them, germline mutations in *CHEK2* found in nine (3.4%) patients outnumbered the most frequent alterations in RCC-associated mutations (7× FH [2.8%]; 3× BAP1 [1.2%]). Consistently, 7/229 (3.1%) mutation carriers with germline *CHEK2* variants were identified among metastatic clear cell renal cancer patients by Ged and colleagues [217]. *CHEK2* germline mutations were also the most frequent alterations found in 19/844 (2.25%) patients with early onset RCC developed before the age of 60 [218]. Notably, among these, second primary cancers (breast, thyroid, colon, blood, and ovarian) were reported in 13 (68%) individuals. With 3/118 (2.5%) individuals suggestive of inherited RCC, *CHEK2* was the second most frequently altered gene (following *BRIP1*) that is not routinely tested for renal cancer predisposition [219]. Unexpectedly, Gadd and colleagues identified germline *CHEK2* variants in 3/117 (2.6%) and 8/651 (1.2%) patients with Wilms tumors in their discovery and validation sets, respectively [220]. Another report by Ciceri et al. [221] found five carriers of rare missense or splicing *CHEK2* variants among 96 Wilms tumor patients from Italy. While evidence of the association of *CHEK2* germline mutations with an increased RCC risk is currently mounting, larger case control studies in RCC patients are required to confirm and refine the magnitude of the associated risk. 

### 3.5. Papillary Thyroid Cancer

A multiple cancer study by Cybulski et al. [20] identified an increased risk of thyroid cancer, particularly in carriers of *CHEK2* truncations (OR = 4.9) and, to a lesser extent, in carriers of p.I157T (OR = 1.9). Observations from this study were confirmed subsequently (Table 4); however, most of the data originate from Poland only and will require confirmation from other populations. 

Recently, Pekova et al. [225] identified pathogenic/likely pathogenic germline *CHEK2* variants in 7/83 (8.4%) Czech pediatric/adolescent patients with papillary thyroid cancer, detecting five (6.0%) p.I157T carriers among them. An interesting report by Zhao and colleagues [226] described a Chinese family with the germline *CHEK2* mutation c.417A>C (p.Y139*; described independently as a recurrent germline mutation in Chinese breast cancer patients [142]) segregating in all four first-degree relatives with papillary thyroid cancer. The authors subsequently analyzed 242 sporadic papillary thyroid cancers and identified two carriers of the p.R180C variant and three carriers of p.H371Y.

Beyond the hereditary cancer genetics, somatic *CHEK2* alterations were characterized as mutations that may contribute to tumor progression in papillary thyroid carcinoma [227].

### 3.6. Colorectal Cancer

Early studies performed by the *CHEK2* consortium (Meijers-Heijboer and colleagues) identified families of c.1100delC carriers with apparently frequent breast and colorectal cancer and denoted this familial cancer cooccurrence as the “hereditary breast *and* colorectal cancer syndrome (HBCC)” [228]. c.1100delC carriers have a strong association with the HBCC phenotype and a trend increasing colorectal cancer risk in HNPCC and HNPCC-like families (Table 5). However, a follow-up study by Nasem and colleagues [229] failed to confirm this finding as they identified only a single c.1100delC carrier in 113 HBCC individuals. Several studies and meta-analyses have focused on colorectal cancer later on and provided evidence of a low-to-moderate risk for c.1100delC and low risk for p.I157T carriers.

Targeted NGS revealed that *CHEK2* was the second most frequently altered gene (following heterozygous *MUTYH*) in the Ambry Genetics ColoNext panel with germline *CHEK2* mutations found in 8/586 colon cancer patients [238]. Subsequent NGS of 450 early-onset colorectal cancer patients from Ohio utilizing a 25-gene panel identified only one *CHEK2* pathogenic mutation; however, the authors reported another 18 *CHEK2* variants as VUS (including six p.I157T) [239]. A recent study of 46 genes in 151 patients with advanced colorectal cancer found 15 carriers of germline mutations. Among them, *CHEK2* with four mutation carriers was the most frequently altered gene [240].

The published data do not provide consistent evidence that germline *CHEK2* alterations substantially contribute to increased colorectal cancer risk. Therefore, the magnitude of colorectal cancer risk needs to be precisely estimated before the formulation of recommendations for a tailored follow-up in mutation carriers. Until then, intensified surveillance may be considered for carriers of *CHEK2* pathogenic mutations from families with multiple appearances of colorectal cancer [241].

### 3.7. Other Cancers

In 2004, Cybulski and colleagues hypothesized that the portfolio of cancers associating with *CHEK2* germline mutations reaches beyond breast and prostate cancers and identified associations with few other cancer types [20]. However, dozens of isolated studies have reported an association of germline *CHEK2* mutations with an increased, decreased, or no risk in particular types of solid cancers. An increased risk has been documented in patients with melanoma [242], endometrial [243,244], or testicular cancer [245]. An association with pancreatic cancer is less evident [20,246,247,248,249], but mutations in genes coding for DDR proteins (including *CHEK2*) in pancreatic cancer patients were associated with improved survival [250,251,252,253]. Germline CHEK2 variants were shown to protect against lung cancer, including in patients with a tobacco-related disease [134,254]. Besides solid tumors, an increased *CHEK2* risk has been reported in patients with hematological malignancies [20,255,256,257,258,259].

Some of the analyzed tumor types are a conundrum evergreen for translational research and a nightmare for clinical geneticists. Ovarian cancer ranks among such recurrently queried tumors. With its poor prognosis and inferior treatment outcomes, it is clinically highly desirable to characterize the predisposition factors enabling tailored surveillance, to prevent and/or detect early ovarian cancer, or to start targeted therapy. All ovarian cancer patients are eligible for germline genetic testing. As ovarian cancer associates with breast cancer, the patients are analyzed by overlapping or identical NGS panels and thus ovarian cancer patients probably represent the largest cancer group explored for mutations in the *CHEK2* gene just after breast cancer patients. However, despite many published results, the association of *CHEK2* mutations with ovarian cancer (or its particular non-high-grade subtypes) can be neither confirmed nor rejected, and it illustrates the stalemate situation with the clinical interpretation of germline *CHEK2* variants [115,260,261,262,263].

## 4. Concluding Remarks and Future Directions

The *CHEK2* gene codes for checkpoint kinase CHK2, activated mainly in DDR. Substrates of activated CHK2 include many intracellular targets regulating numerous signaling pathways. However, the quantitative importance of CHK2 in these regulations in particular human tissues remains to be identified.

Pathogenic germline *CHEK2* mutations rank among the most frequent alterations in various tumors. The association of germline *CHEK2* variants has been confirmed for the most frequent gender-specific tumors, including breast and prostate. Despite the high probability of an association with several other cancers, including renal and thyroid cancer, there is no recommendation to prevent these tumors in *CHEK2* carriers. Unfortunately, an association with numerous tumor types and subtypes remains uncertain so far. The major sources of this uncertainty include insufficient numbers of patients with a comprehensive *CHEK2* mutation analysis (including CNV), deficient functional classification of *CHEK2* VUS, and a lack of a precise use of geographically matched population controls for unequivocal evaluation of the association. The latter point disregarded by many studies is of particular importance if we assume a large population diversity of germline *CHEK2* mutations worldwide, a high frequency of its germline alterations (comparable with that of *ATM* for which the coding sequence is five times larger than that of the *CHEK2* gene), and incomplete penetrance of *CHEK2* mutations.

## Figures and Tables

**Table 1:** Analyses of the entire CHEK2 coding sequence (separately or as part of panel next-generation sequencing (NGS)) or analyses of specific variant(s) in breast cancer (BC) patients.

Reference	Population	P: Patients C: Controls	Analysis *	Odds Ratio (95% Confidence Interval); p—Remark (Statistically Insignificant in *Italics*)
*Female breast cancer*				
Fostira 2020 [146]	GR	P: 1382 high-risk BC patients C: ExAC/FLOSSIES	*CHEK2* (panel NGS)	*1.7 (0.98–2.7); 0.11—all LoF variants/ExAC* 2.6 (1.44–4.68); 0.003—all LoF variants/FLOSSIES 3.8 (1.86–7.12); 1.2 × 10^−3^—missense deleterious/ExAC 5.9 (2.38–14.8); 1.2 × 10^−4^—missense deleterious/FLOSSIES
Kurian 2020 [147]	US (66% white)	P: 2,195 postmenopausal BC C: 2322 age-matched PMC	*CHEK2* (panel NGS)	N.D.; *CHEK2* PV found in 0.59% P and 0.26% C
Rogoza-Janiszewska 2020 [148]	PL	P. 2,464 BC diagnosed at <41 C: from Cybulski 2019	c.1100delC; c.444+1G>A; del5395	3.8 (2.53–5.58); <0.0001—BC at < 41 y; all truncations 4.6 (2.44–8.80); <0.0001—BC at < 31 y; all truncations
Kleiblova 2019 [115]	CZ	P: 1526 high-risk female BC C: 3360 PMC	*CHEK2* (panel NGS)	7.94 (3.90–17.47); 4.1 × 10^−11^—unilat. BC: truncations 3.90 (1.24–13.35); 0.009—unilat. BC: deleterious missense 8.39 (1.92–28.74); 0.003—bilat. BC: truncations *3.77 (0.08–31.42); 0.26—bilat BC: deleterious missense*
Cybulski 2019 [149]	PL	P: 1,018 hereditary BC C: 4346 PMC	c.1100delC c.444+1G>A del5395	6.9 (3.2–14.7); <0.0001—for c.1100delC 8.4 (3.0–23.3); <0.0001—for c.444+1G>A 6.5 (3.2–13.4); <0.0001—for del53957.2 (4.5–11.6); <0.0001—for all above truncations
Nurmi 2019 [150]	FI	P: 3156 BC C: 2089 PMC	c.319+2T>A; c.444+1G>A; c.1100delC	5.40 (1.58–18.45); 0.007—for c.319+2T>A unselected BC 6.04 (1.65–22.10); 0.007—for c.319+2T>A familial BC
Girard 2019 [151]	FR	P: 1207 *BRCA1/2*^−ve^ BC pts having sister with BC C: 1199 non-cancer PMC	*CHEK2* (WES + panel NGS)	3.0 (1.9–5.0); 1 × 10^−5^—any rare variant 5.8 (2.0–16.9); 0.001—LoF variants 2.4 (1.4–4.3); 0.002—likely-deleterious missense
Hauke 2018 [126]	DE	P: 5589 *BRCA1/2*^−ve^ BC C: 2189 non-cancer PMC	*CHEK2* (panel NGS)	3.72 (1.99–6.94); <0.0001—truncations
Momozawa 2018 [145]	JP	P: 7051 BC C: 11,241 PMC	*CHEK2* (panel NGS)	3.2 (1.6–6.8); 3.2 × 10^−4^
Decker 2017 [152]	UK	P: 13,087 BC C: 5488 PMC	*CHEK2* (& 3 other genes)	3.11 (2.15–4.69); 5.6 × 10^−11^—truncations *1.36 (0.99–1.87); 0.066*—*all rare missense* 1.51 (1.02–2.24); 0.047—rare missense in any domain 3.27 (1.66–5.83); 0.0014—bilateral BC 3.42 (2.33–5.21); 1.5 × 10^−11^—ER^+ve^ BC 3.98 (2.62–6.21)—age at dg < 50 years 3.37 (2.24–5.22)—age at dg = 50–60 years 2.12 (1.35–3.41)—age at dg > 60 years
Slavin 2017 [153]	US (80% white)	P: 2266 *BRCA1/2*^−ve^ fam. BC C: ExAC	*CHEK2* (panel NGS)	1.62 (1.03–2.51); 0.004 – truncations
Couch 2017 [154]	US (white)	P: 29,090 BC C: 25,215 ExAC-NFE	*CHEK2* (panel NGS)	2.31 (1.88–2.85); 3.04 × 10^−17^—c.1100delC 2.26 (1.89–2.72); 1.75 × 10^−20^—PVs (w/o p.I157T, p.S428F) 1.48 (1.31–1.67); 1.11 × 10^−10^—any var (w p.I157T, p.S428F) 1.35 (1.1–1.63); 0.0002; bilateral BC
Schmidt 2016 [155]	BCAC	P: 44,777 population+ hospital-based BC C: 42,977 PMC	c.1100delC	2.26 (1.90–2.69); 2.3×10^−20^—invasive BC 2.55 (2.10–3.10); 4.9 × 10^−21^—ER^+ve^ BC *1.32 (0.93–1.88); 0.12*—*ER^−ve^ BC*
Naslund-Koch 2016 [156]	DK	2442 BC pts /86,975 individ. (longitudinal study);	c.1100delC	2.08 (1.51–2.85); <0.001
Southey 2016 [157]	BCAC	P: 42,671 C: 42,164 PMC	iCOGS array incl. 6 rare *CHEK2* variants	2.26 (1.29–3.95); 0.003—for p.R117G 1.33 (1.05–1.67); 0.016—for p.R180C 1.70 (0.73–3.93); 0.210—for p.E239K 5.06 (1.09–23.5); 0.017—for p.R346C *1.03 (0.62–1.71); 0.910*—*for p.D438Y*
Liu Y 2011 [158]	CN (Han)	P: 118 familial BC P: 909 unselected BC C: 1228 healthy PMC	*CHEK2* (dHPLC) for familial BC	5.99 (1.98–18.11); 0.002—for p.H371Y familial BC 2.43 (1.07–5.52); 0.034—for p.H371Y unselected BC
Cybulski 2011 [159]	PL	P: 7494 *BRCA1*^−ve^ BC C: 4346 PMC	c.1100delC; c.444+1G>A; del5395	3.6 (2.6–5.1)—all BC 3.3 (2.3–4.7)—patients with no BC family history 5.0 (3.3–7.6)—patients with BC in 1° or 2° relative 7.3 (3.2–16.8)—patients with BC in 1° and 2° relatives
Desrichard 2011 [122]	FR	P: 507 *BRCA1/2*^−ve^ BC C: 513 non-cancer PMC	*CHEK2* (sequencing)	4.15 (1.38–12.50); 0.007—any variant 5.18 (1.49–18.00); 0.004—deleterious (p.K244R ex)
Le Calvez-Kelm 2011 [160]	US/CA/AU	P: 1242 BC ≤ 45y C: 1109 non-ca PMC female	*CHEK2* (HRM)	6.18 (1.76–21.8)—truncations/splice mutations 2.20 (1.20–4.01)—rare missense
Fletcher 2009 [161]	UK/FI/NL/ RU/DE	P: 1828 bilateral BC C: 7030 PMC	c.1100delC	6.43 (4.33–9.53); <0.0001—second primary for mut. carriers
Weischer 2007 [162]	DK	P + C: 9231 (prospective) P: 1101 BC/4665 PMC (case-control)	c.1100delC	3.2 (1.0–9.9)—BC (prospective study) 2.6 (1.3–5.4)—BC (case-control study)
Cybulski 2006 [163]	PL	P: 3228 BC diagnosed at ≤50 C: 5496 PMC	c.1100delC c.444+1G>A p.I157T	2.3 (1.1–4.8); 0.04—for c.1100delC 2.4 (1.4–4.2); 0.002—for c.444+1G>A 2.4 (1.5–3.7); 0.0001—for any truncation 1.4 (1.1–1.6); 0.002—for p. I157T
Chekmariova 2006 [164]	RU	P: 660 unilat; 155 bilat BC C: 448 middle aged females;	c.1100delC (ASO PCR)	9.8 (1.34–198.26); 0.007 - early onset/bilat BC/C carriers frequencies: 3.4/5.2/0.2%
Cybulski 2004 [20]	PL	P: 1017 BC C: 4000 PMC	c.1100delC; c.444+1G>A; p.I157T	2.2; *p* = 0.02—for c.1100delC and c.444+1G>A 1.4; *p* = 0.02—for p.I157T
Caligo 2004 [130]	IT	P: 939 BC (incl. *BRCA1/2*^+ve^) C: 334 PMC	c.1100delC	*N.S.; frequency of carriers 0.11% (95% CI 0.00–0.59%)*
Dufault 2004 [165]	DE	P: 516 *BRCA1/2*^−ve^ BC C: 500 PMC (1,315 PMC for c.1100delC)	*CHEK2*	3.44 (1.19–9.95); 0.016—c.1100delC 3.9 (1.3–10.9)—c.1100delC and c.1214del4
CHEK2 BC consortium 2004 [166]	UK/NL/FI/ DE/AU	P: 10,860 BC C: 9065 multinatl.	c.1100delC	2.34(1.72–3.20); 1 × 10^−7^—all BC 2.23 (1.60–3.11)—BC w/o BC in 1° relative 3.12 (1.90–5.15)—BC with 1 BC in 1° relative 4.17 (1.26–13.75)—BC with ≥2 BC in 1° relatives
CHEK2 BC consortium 2002 [167]	UK/NL/ US/CA	P: 636 unselected BC P: 718 *BRCA1/2*^-ve^ BC C: 1620 multinatl.	c.1100delC	*2.52 (0.78–8.18)*—*unselected BC* 1.70 (1.32–3.38)—*BRCA1/2*^−ve^ BC
Vahteristo 2002 [110]	FI	P: 1035 unselected BC C: 1885 PMC (blood donors)	c.1100delC	*1.48 (0.83–2.65); 0.182*—*unselected BC* 2.27 (1.11–4.63); 0.021—familial BC 6.17 (1.87–20.32); 0.007 bilat. vs. unilat. BC
*Male breast cancer*				
Kleiblova 2019 [115]	CZ	P: 48 male BC C: 3360 PMC	*CHEK2* (panel NGS)	20.21 (3.50–80.00); 8.6 × 10^−4^—truncations *11.87 (0.25–100.83); 0.1*—*deleterious missense*
Liang 2018 [168]	meta	P: 1063 male BC C: 31,571	c.1100delC	3.13 (1.94–5.07)
Hallamies 2017 [169]	FI	P: 68 male BC C: 1885 from [110]	c.1100delC	4.47 (1.51–13.18); 0.019
Wasielewski 2009 [170]	NL	P: 71 male BC C: 1692	c.1100delC	4.1 (1.2–14.3); 0.05
CHEK2 consortium 2002 [167]	UK/NL/US/CA	P: 52 male BC families C: 1620 multinatl.	c.1100delC	10.28 (3.54–29.87)
*Meta-analyses*				
Yang 2019 [171]	BCAC+ ABCC meta	P: 122,977 + 24,206 BC C: 105,974 + 24,775 PMC	p.I157T	1.28 (1.17–1.39); 9.66 × 10^−9^—for Europeans only 1.35 (1.18–1.54); 9.82 × 10^−6^—for ER^+ve^ BC *0.95 (0.81–1.12); 0.55*—*for ER^−ve^ BC*
Liang 2018 [168]	meta	P: 118,735 BC C: 195,807	c.1100delC	2.88 (2.65–3.22)—female BC 2.87 (1.85–4.47)—early-onset BC 3.21 (2.41–4.29)—familial BC 3.13 (1.94–5.07)—male BC
Aloraifi 2015 [113]	meta	P. 7283 C: 13,785	*CHEK2* truncations	3.25 (2.55–4.13)
Han 2013 [172]	meta	P: 15,985 BC C: 18,609	p.I157T	1.58 (1.42–1.75); <0.0001
Liu 2012 [173]	meta	P: 19,621 BC C: 27,001	p.I157T	1.48 (1.31–1.68); <0.0001—unselected BC 1.48 (1.16–1.89); <0.0001—familiar BC 1.47 (1.29–1.66); <0.0001—early onset BC 4.17 (2.89–6.03); <0.0001—lobular BC
Yang 2012 [174]	meta	P: 29,154 BC C: 37,064	c.1100delC	2.33 (1.79–3.05)—unselected BC 3.72 (2.61–5.31)—familiar BC 2.78 (2.28–3.39)—early onset BC
Zhang 2011 [175]	meta	P: 9970/C:7526 P: 13,331/C: 10,817 P: 10,543/C:10,817 P: 41,791/C: 50,910	c.444+1G>A del5395 c.1100delC p.I157T	3.07 (2.03–4.63); 9.82 × 10^−8^—for variant c.444+1G>A 2.53 (1.61–3.97); 6.33 × 10^−5^—for variant del5395 3.10 (2.59–3.71); <10^−20^—for variant c.1100delC 1.52 (1.31–1.77); 4.76 × 10^−8^—for variant p.I157T
Weischer 2008 [176]	meta	P: 26,488 C: 27,402	c.1100delC	2.7 (2.1–3.4)—unselected BC 2.6 (1.3–5.5)—early onset BC 4.8 (3.3–7.2)—familial BC

* *CHEK2* = an analysis of the entire coding sequence (dominantly without copy number variations (CNV)); otherwise specified if certain *CHEK2* variants were genotyped. AU—Australia; ABCC—Asian Breast Cancer Consortium; BC—breast cancer; BCAC—Breast Cancer Association Consortium; CA—Canada; CN—China; CZ—Czech Republic; DE—Germany; DK—Denmark; ES—Spain; EU—European Union; ExAC—Exome Aggregation Consortium; FI—Finland; FLOSSIES—Fabulous Ladies Over Seventy; FR—France; meta—meta-analysis; GR—Greece; IT—Italy; LoF—loss-of-function; JP—Japan; NL—Netherlands; PL—Poland; PMC—population-matched control; RU—Russia; US—the USA. The analyses that failed to demonstrate an association are shown in italics.

**Table 2:** Analyses of the entire *CHEK2* coding sequence (separately or as part of panel NGS) or analyses of specific variant(s) in prostate cancer (PrC) patients.

Reference	Population	P: Patients C: Controls	Analysis **	Odds Ratio (95% Confidence Interval); p—Remark (Statistically Insignificant in *Italics*)
Brandao 2020 [202]	PT PRACTICAL	P: 462 early-onset/familial PrC C: 710 PMC P: 55,162 PrC/C: 36,147	c.349A>G (p.R117G)	*7.7 (0.9–66.6); 0.06—PT PrC p.R117G* 1.9 (1.1–3.2); 0.04—PRACTICAL PrC p.R117G
Momozawa 2018 [144]	JP	P: 7636 C: 12,366	Panel NGS (8 genes)	*2.43 (0.91–6.86); 0.06*
Conti 2017 [203]	AAPC, GH	AAPC–P:4,853 PrC; C: 4678 GH–P: 474; C: 458	GWAS array (rs78554043 = rs17886163; *CHEK2* c.1343T>G; p.I448S)	1.60 (1.27–2.00); 5.02 × 10^−5^—for AAPC PrC 2.45 (1.33–4.52); 0.004—for Ghana PrC
Naslund-Koch 2016 [156]	DK	86,975 individuals (longitudinal study); 1340 developed PrC	c.1100delC	*1.60 (1.00–2.56); 0.05*
Southey 2016 [157]	OCAC	P: 22,301 PrC C: 22,320 PMC	iCOGS array incl. 6 rare *CHEK2* variants	*1.46 (0.71–3.02); 0.3—for p.R117G* *1.02 (0.73–1.44); 0.9—for p.R180C* *1.47 (0.41–5.35); 0.6—for p.E239K* *1.07 (0.28–4.07); 0.9—for p.D438Y* 2.21 (1.06–4.63); 0.03—for p.D438Y 3.03 (1.53–6.03); 0.001—for I448S in Africans
Pritchard 2016 [118]	US, UK	P: 692 metastat. PrC C: ExAC/TCGA	Panel NGS	3.1 (1.5–5.6); 0.002—vs. ExAC (excl. p.I157T) 4.7 (2.2–8.5); <0.001—vs. TCGA (excl. p.I157T)
Wang 2015 [204]	meta	P: 6409 PrC C: 11,634	c.1100delC c.444+1G>A p.I157T	3.29 (1.85–5.85); <0.001—c.1100delC *1.59 (0.79–3.20); 0.20—c.1100delC, familial* *1.58 (0.93–2.71); 0.09—c.444+1G>A* 1.80 (1.51–2.14); <0.001—p.I157T
Hale 2014 [205]	meta	P: 5,124 PrC C: 9,258	c.1100delC	1.98 (1.23–3.18); 0.004—unselected 3.39 (1.78–6.47); 0.0001—familial
Cybulski 2006 [206]	PL	P: 1864 PrC (incl. 249 famil.) C: 5496	c.1100delC; c.444+1G>A; 5395del; p.I157T	2.3 (1.1–3.9); <0.001—truncations, sporadic 4.7 (2.5–9.0); <0.001—truncations, familial 1.6 (1.3–2.0); <0.001—p.I157T, sporadic 2.7 (1.8–4.1); <0.001—p.I157T, familial
Weischer 2007 [162]	DK	P: 116 PrC (prospective) C: 3999 PMC men (prospect.)	c.1100delC	*2.3 (0.6–9.5) PrC prospective study*
Johnson 2005 [177]	UK	P: 469 bilat. BC	c.1100delC	2.41 (1.67–3.36)—risk of PrC for relatives of patients with bilateral BC
Cybulski 2004 [20]	PL	P: 690 PrC C: 4000 PMC	c.1100delC; c.444+1G>A; p.I157T	2.2; 0.04—truncations 1.7; 0.002—p.I157T
Seppala 2003 [207]	FI	P1: 537 unselected PrC; P2: 120 hereditary PrC C: 510 non-PrC men	*CHEK2* (SSCP: heredit. PrC) c.1100delC/p.I157T	*3.14 (0.65–15.16); 0.15—c.1100delC, sporadic* 8.24 (1.49–45.54); 0.02—c.1100delC, hereditary *1.48 (0.89–2.46); 0.13—p.I157T, sporadic* 2.12 (1.06–4.27); 0.04—p.I157T, hereditary
Dong 2003 [200]	US	P1: 400 sporadic PrC; P2: 298 familial PrCC: 510 non-PrC men	*CHEK2* (DHPLC)	2.71 (1.04–7.04); 0.049 *—sporadic PrC 2.66 (0.98–7.28); 0.078 *– familial PrC 6.84 (0.86–54.1); 0.05 *—sporadic (w/o p.I157T) 5.74 (0.64–51.5); 0.17 *—familial (w/o p.I157T)

* Calculated using WINPEPI [208]; ** *CHEK2* = an analysis of the entire coding sequence (dominantly without CNV); otherwise specified if certain *CHEK2* variants were genotyped. AAPC—African Ancestry Prostate Cancer; DK—Denmark; ES—Spain; ExAC—Exome Aggregation Consortium; FI—Finland; GH—Ghana; meta—meta-analysis; JP—Japan; OCAC—Ovarian Cancer Association Consortium; PL—Poland; PT—Portugal; PMC—population-matched control; PRACTICAL - The Prostate Cancer Association Group to Investigate Cancer Associated Alterations in the Genome; TCGA - The Cancer Genome Atlas; US—the USA. The analyses that failed to demonstrate an association are shown in italics.

**Table 3:** Analyses of the entire *CHEK2* coding sequence (separately or as a part of panel NGS) or analyses of specific variant(s) in renal cell carcinoma (RCC) patients.

Reference	Population	P: Patients C: Controls	Analysis *	Odds Ratio (95% Confidence Interval); p—Remark (Statistically Insignificant in *Italics*)
Zlowocka-Perlowska 2019 [214]	PL	P: 835 invasive RCC C: 8304 non-cancer	c.1100delC; c.444+1A>G; 5395del; c.I157T	2.5 (1.5–4.1); 0.0003—for truncations 2.0 (1.6–2.6); <0.001—for p.I157T
Carlo 2018 [215]	US	P: 254 RCC (stage III-IV) C: ExAC	*CHEK2*	3.0 (1.3–5.8); 0.003
Ge 2016 [216]	GWAS	P: 1322 C: 3428	p.I157T	0.63 (0.44–0.89); 0.01
Naslund-Koch 2016 [156]	DK	138/86,975 individuals developed RCC	c.1100delC	3.61 (1.33–9.79); 0.01
Weischer 2007 [162]	DK	P: 33 RCC (prospective) C: 9166 PMC (prospect.)	c.1100delC	9.8 (2.3–41.2) RCC prospective study
Cybulski 2004 [20]	PL	P: 264 RCC C: 4000 PMC	c.1100delC; c.444+1G>A; p.I157T	*1.0*; *p = 0.8—truncations* 2.1; *p* = 0.0006—for p.I157T

* *CHEK2* = an analysis of the entire coding sequence (dominantly without CNV); otherwise specified if certain *CHEK2* variants were genotyped. DK—Denmark; GWAS—genome-wide association study; PL—Poland; US—the USA. The analyses that failed to demonstrate an association are shown in italics.

**Table 4:** Analyses of the *CHEK2* variants in Polish (PL) patients with papillary thyroid cancer (PTC).

Reference	Population	P: Patients C: Controls	Analysis of Specific *CHEK2* Variants	Odds Ratio (95% Confidence Interval); p—Remark (Statistically Insignificant in *Italics*)
Kaczmarek-Rys 2015 [222]	PL	P: 602 differentiated PTC C: 829 PMC	p.I157T	1.81 (1.20–2.72); 0.004—p.I157T heterozygote 12.81 (0.6–248.46); 0.02—p.I157T homozygote
Siolek 2015 [223]	PL	P: 468 unselected PTC C: 468 matched non-cancer	c.1100delC; c.444+1G>A; 5395del; p.I157T	5.7 (1.7–19.3); 0.006—truncations 2.8 (1.7–4.6); 0.0001—p.I157T
Wojcicka 2014 [224]	PL	P: 1781 PTC C: 2081 healthy PMC	p.I157T	2.21 (1.69–2.88); 2.37 × 10^−10^—for p.I157T
Cybulski 2004 [20]	PL	P: 173 PTC C: 4000 PMC	c.1100delC; c.444+1G>A; p.I157T	4.9; 0.006—truncations 1.9; 0.04—p.I157T

**Table 5:** Analyses of the entire *CHEK2* coding sequence (separately or as a part of panel NGS) or analyses of specific variant(s) in colorectal cancer (CRC) patients.

Reference	Population	P: Patients C: Controls	Analysis	Odds Ratio (95% Confidence Interval); p—Remark (Statistically Insignificant in *Italics*)
Xiang 2017 [230]	meta	Revision of the analysis from [231]		1.8 (1.2–2.7)—unselected CRC *2.4* (*0.9–6.6*)—*familial CRC* *1.7* (*0.9–2.9*)–*sporadic CRC*
Naslund-Koch 2016 [156]	DK	1131/86,975 individuals developed CRC	c.1100delC	*0.86* (*0.43–1.72*);*0.68*
Ma 2014 [232]	meta	P: 3874 CRC/ C:11,630 P: 6042 CRC/ C:17,051	c.1100delC p.I157T	1.88 (1.29–2.73); 0.001 1.56 (1.32–1.84); 1.22 × 10^−7^
Han 2013 [172]	meta	P: 3166 CRC C: 9844	p.I157T	1.67 (1.24–2.26); 0.0008
Liu 2012 [233]	meta	P: 4029 CRC C: 13,844	p.I157T	1.61 (1.40–1.87); <0.001—unselected CRC 1.48 (1.23–1.77); <0.001—sporadic CRC 1.97 (1.41–2.74); <0.001—familial CRC
Xiang 2011 [231]	meta	P: 4,194 CRC C: 10,010	c.1100delC	2.11 (1.41–3.16); 0.0003—unselected CRC 2.80 (1.74–4.51); <0.0001—familial CRC *1.45 (0.49–4.30); 0.5*—*sporadic CRC*
Suchy 2010 [234]	PL	P: 463 HNPCC-related C: 5496 PMC	c.1100delC; c.444+1G>A; del5395; p.I157T	*1.0* (*0.4*–*2.6*); *1.0*—*truncations* 1.7 (1.2–2.4); 0.007—p.I157T
Kleibl 2009 [235]	CZ	P: 631 CRC C: 683 PMC	FHA-coding region c.1100delC;5395del	2.3 (1.3–4.0); 0.003—all variants in FHA domain 2.0 (1.1–3.6); 0.03—p.I157T only *2.3* (*0.4–12.8*); *0.4*—*c.1100delC; zero 5395del carriers*
Weischer 2007 [162]	DK	P: 210 (prospective) C: 9007 PMC (prospect.)	c.1100delC	*1.6 (0.4–6.5)*—*prospective CRC*
Cybulski 2007 [236]	PL	P. 1085 unselected CRC C: 5496 controls	c.1100delC; c.444+1G>A; del5395; p.I157T	*1.0 (0.5–1.8); 0.9*—*truncations* 1.5 (1.2–2.0); 0.002—p.I157T
Djureinovic 2006 [237]	SE	P: 174 familial CRC P: 644 unselected CRC C: 760 PMC	c.1100delC	*1.76 (0.34–9.09); 0.6*—*familial CRC* *1.42 (0.43–4.67); 0.7*—*sporadic CRC*
Irmejs 2006 [133]	LV	P: 235 C: 978 newborn PMC	p.I157T	1.7 (1.01–2.70); *p* < 0.05
Meijers-Heijboer 2003 [228]	UK/NL/ US/CA	P: 329 CRC C: 1620 [167]	c.1100delC (ASO)	*2.34 (0.95–5.79); 0.07*—*HNPCC-like families* 5.19 (2.17–12.4); < 0.001—HBCC families

CZ—Czech Republic; DK—Denmark; LV—Latvia; NL—the Netherlands; PL—Poland; SE—Sweden; UK—the United Kingdom, US—the USA. The analyses that failed to demonstrate an association are shown in italics.
